# Feasibility and reproducibility of semi-automated longitudinal strain analysis: a comparative study with conventional manual strain analysis

**DOI:** 10.1186/s12947-023-00309-5

**Published:** 2023-07-19

**Authors:** Gui-juan Peng, Shu-yu Luo, Xiao-fang Zhong, Xiao-xuan Lin, Ying-qi Zheng, Jin-feng Xu, Ying-ying Liu, Li-xin Chen

**Affiliations:** Shenzhen Medical Ultrasound Engineering Center, Department of Ultrasound, Shenzhen People’s Hospital (The Second Clinical Medical College, Jinan University; The First Affiliated Hospital, Southern University of Science and Technology), Shenzhen, 518020 China

**Keywords:** Strain, Speckle tracking echocardiography, Automatic assessment, Left ventricle, Right ventricle

## Abstract

**Background:**

Conventional approach to myocardial strain analysis relies on a software designed for the left ventricle (LV) which is complex and time-consuming and is not specific for right ventricular (RV) and left atrial (LA) assessment. This study compared this conventional manual approach to strain evaluation with a novel semi-automatic analysis of myocardial strain, which is also chamber-specific.

**Methods:**

Two experienced observers used the AutoStrain software and manual QLab analysis to measure the LV, RV and LA strains in 152 healthy volunteers. Fifty cases were randomly selected for timing evaluation.

**Results:**

No significant differences in LV global longitudinal strain (LVGLS) were observed between the two methods (-21.0% ± 2.5% vs. -20.8% ± 2.4%, *p* = 0.230). Conversely, RV longitudinal free wall strain (RVFWS) and LA longitudinal strain during the reservoir phase (LASr) measured by the semi-automatic software differed from the manual analysis (RVFWS: -26.4% ± 4.8% vs. -31.3% ± 5.8%, *p < *0.001; LAS: 48.0% ± 10.0% vs. 37.6% ± 9.9%, *p < *0.001). Bland–Altman analysis showed a mean error of 0.1%, 4.9%, and 10.5% for LVGLS, RVFWS, and LASr, respectively, with limits of agreement of -2.9,2.6%, -8.1,17.9%, and -12.3,33.3%, respectively. The semi-automatic method had a significantly shorter strain analysis time compared with the manual method.

**Conclusions:**

The novel semi-automatic strain analysis has the potential to improve efficiency in measurement of longitudinal myocardial strain. It shows good agreement with manual analysis for LV strain measurement.

**Graphical Abstract:**

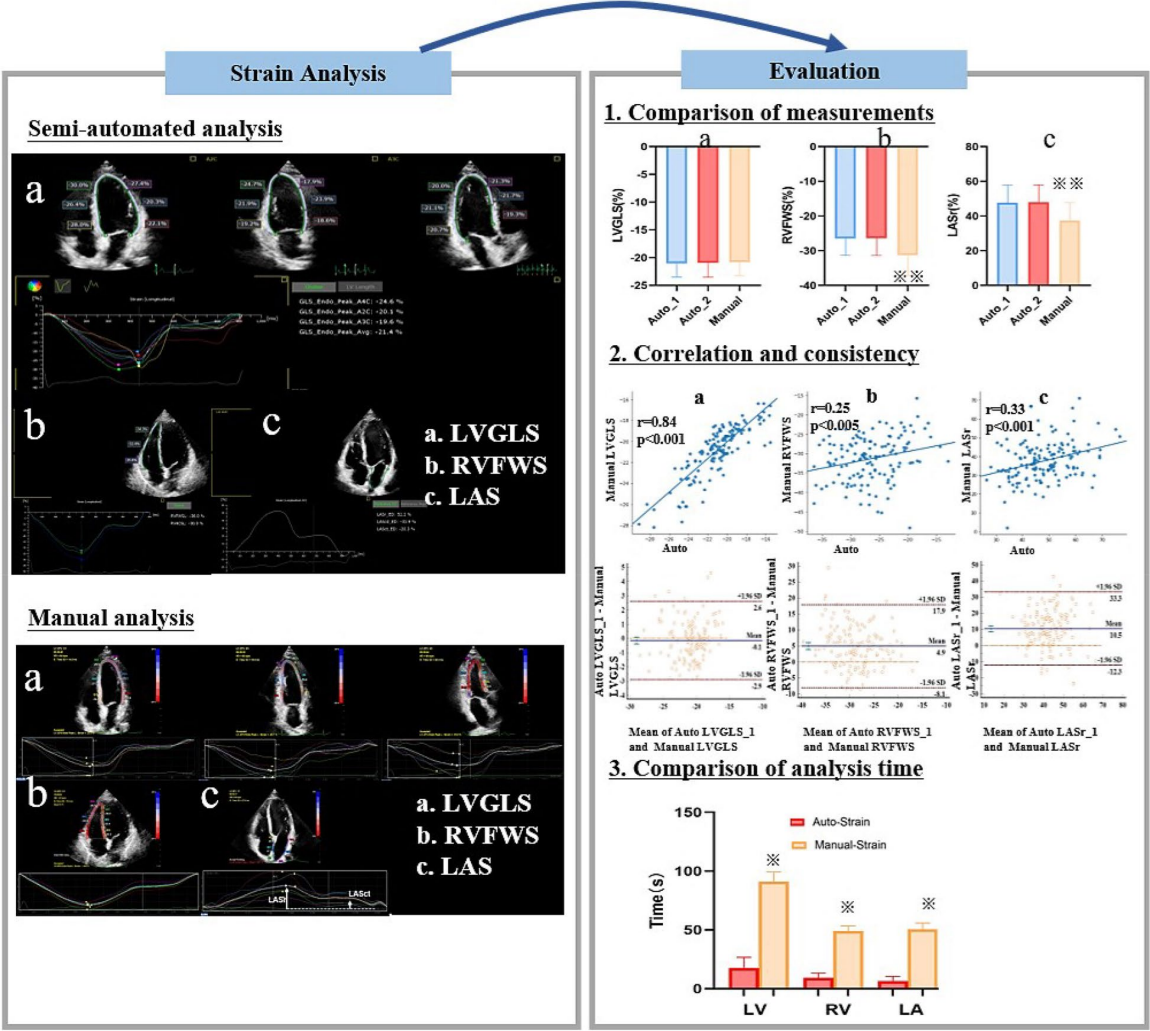

**Supplementary Information:**

The online version contains supplementary material available at 10.1186/s12947-023-00309-5.

## Introduction

Myocardial function measurement is the basis for the diagnosis of cardiac diseases. Today it can perform using two-dimensional speckle-tracking echocardiography (2D-STE), which is a technique designed to evaluate myocardial deformation. Left ventricular global longitudinal strain (LVGLS) is the most mature and widely used parameter obtained using 2D-STE. LVGLS has been shown to have additional value in risk stratification and outcome prediction compared to LV ejection fraction (LVEF) in a variety of clinical conditions [1–5]. The 2015 guidelines of the European Society of Cardiovascular Imaging (EACVI) and the American Society of Echocardiography (ASE) recommended the use of LVGLS as a supplement to the LVEF when assessing LV function [6]. Although 2D-STE was originally used as a tool specifically designed for LV strain measurement, researchers also applied it to the analysis of right ventricular (RV) and left atrial (LA) myocardial deformation [7–9]. Several studies have shown that outcome prediction can be improved in many heart diseases using LA strain [7, 10–12] and RVGLS [13–16].

Challenges remain in the application of 2D-STE in clinical practice. First, because the shape, structure, and function of the LA and RV are different from those of the LV, application of the same 2D-STE technique to strain analysis of different cardiac chambers is controversial [7]. Second, tedious and time-consuming manual editing of non-automated myocardial strain evaluations limits clinical application of conventional echocardiographic techniques for strain analysis [17]. Therefore, automatic, and chamber-specific methods for myocardial strain assessment have been recently introduced and used in research [18].

At present, there are few comparative studies between methods for automatic and conventional manual strain analysis. Thus, the purpose of this study was to compare AutoStrain, a new automatic and chamber-specific strain analysis software (referred to as automatic strain analysis), with the conventional QLab software for LV strain analysis (referred to as manual strain analysis).

## Methods

### Study cohort

From March 2021 to April 2021, a total of 159 consecutive healthy volunteers who underwent routine annual check-up in our hospital were recruited. For all the volunteers, the ECG and X-ray results were normal. All subjects provided written informed consent and study was approved by the Human Research Ethics Committee of Shenzhen People’s Hospital.

### Echocardiography

A Philips Epic7C or Philips CVx cardiac ultrasound scanner (Philips®, Best, The Netherlands) was used to acquire the standard apical four-chamber, two-chamber, and long-axis views as well as the RV-focused four-chamber view of the heart. At least 4 cardiac cycles were recorded for each view. Images were saved in DICOM format. LV end-diastolic volume (LVEDV), end-systolic volume (LVESV), and LVEF were calculated by the biplane Simpson’s method.

### Strain analysis

The LV strain analysis was performed in the standard apical four-chamber, two-chamber, and long-axis view according to the guidelines of the ASE [19]. The RV-focused four-chamber view was used for the RV strain analysis, and the standard apical four-chamber view for the LA analysis [20]. The same image cine-loops were used for online analysis by the AutoStrain and offline analysis by the Qlab 9.1 software (Philips Medical Systems). Two experienced observers (G.P. and S.L., with more than 5 to 7 years-experience in echocardiography) performed the strain evaluations blinded to each other.

### Automated strain analysis

**Fig. 1:**
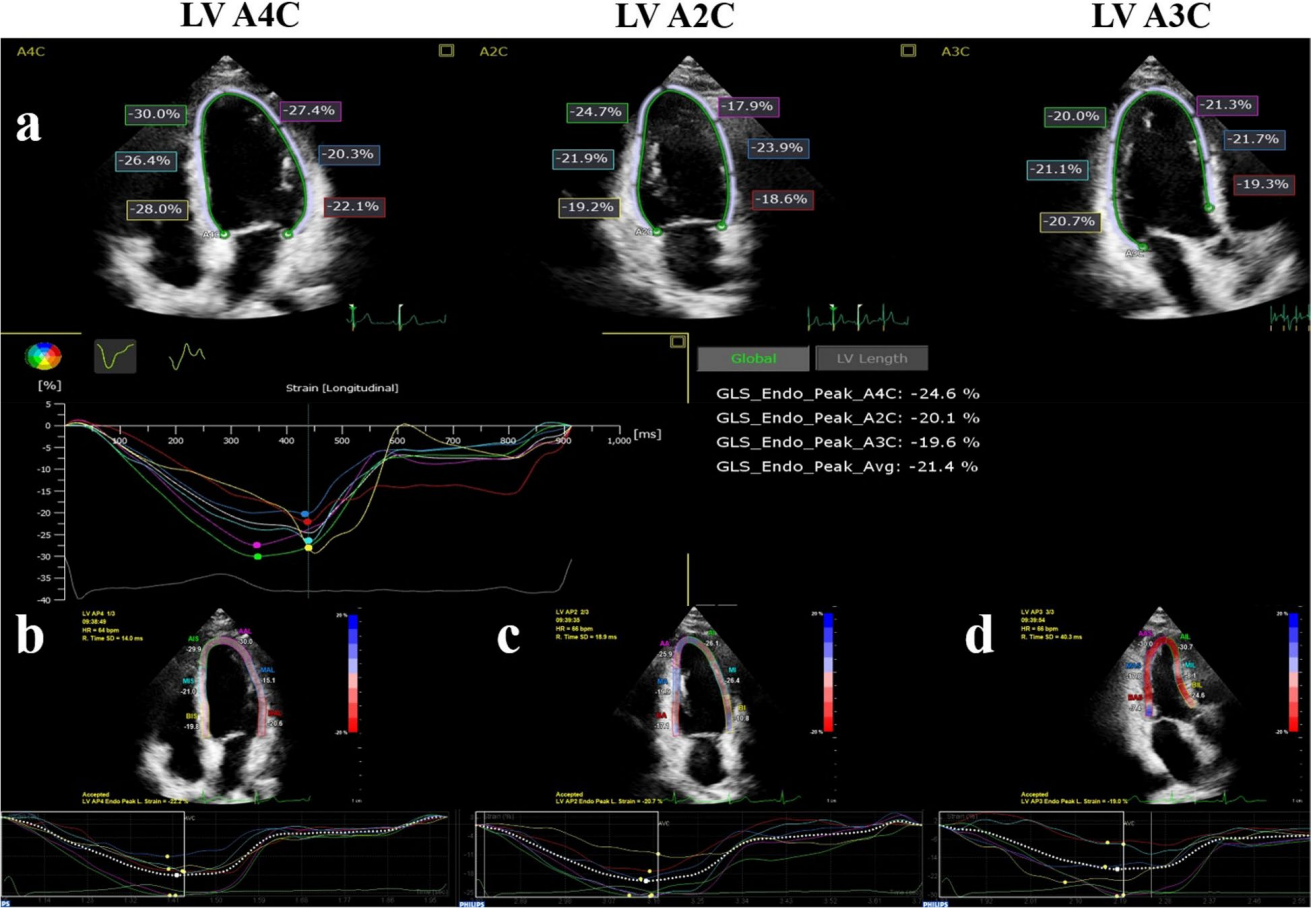
Evaluation of left ventricular global longitudinal strain (LVGLS) by the semi-automated (**a**) and manual (**b**-**d**) software for strain analysis. **a**: The semi-automatic analysis software automatically recognizes, segments and tracks the LV myocardium of the three standard apical views (four-chamber, two-chamber and three-chamber view), and calculates the longitudinal strain of each view and GLS. **b**-**d**: After manually drawing the location of the apex and the medial and lateral annulus, the software tracks the LV myocardium frame by frame. The strain and strain curves of each apical view are shown

**Fig. 2:**
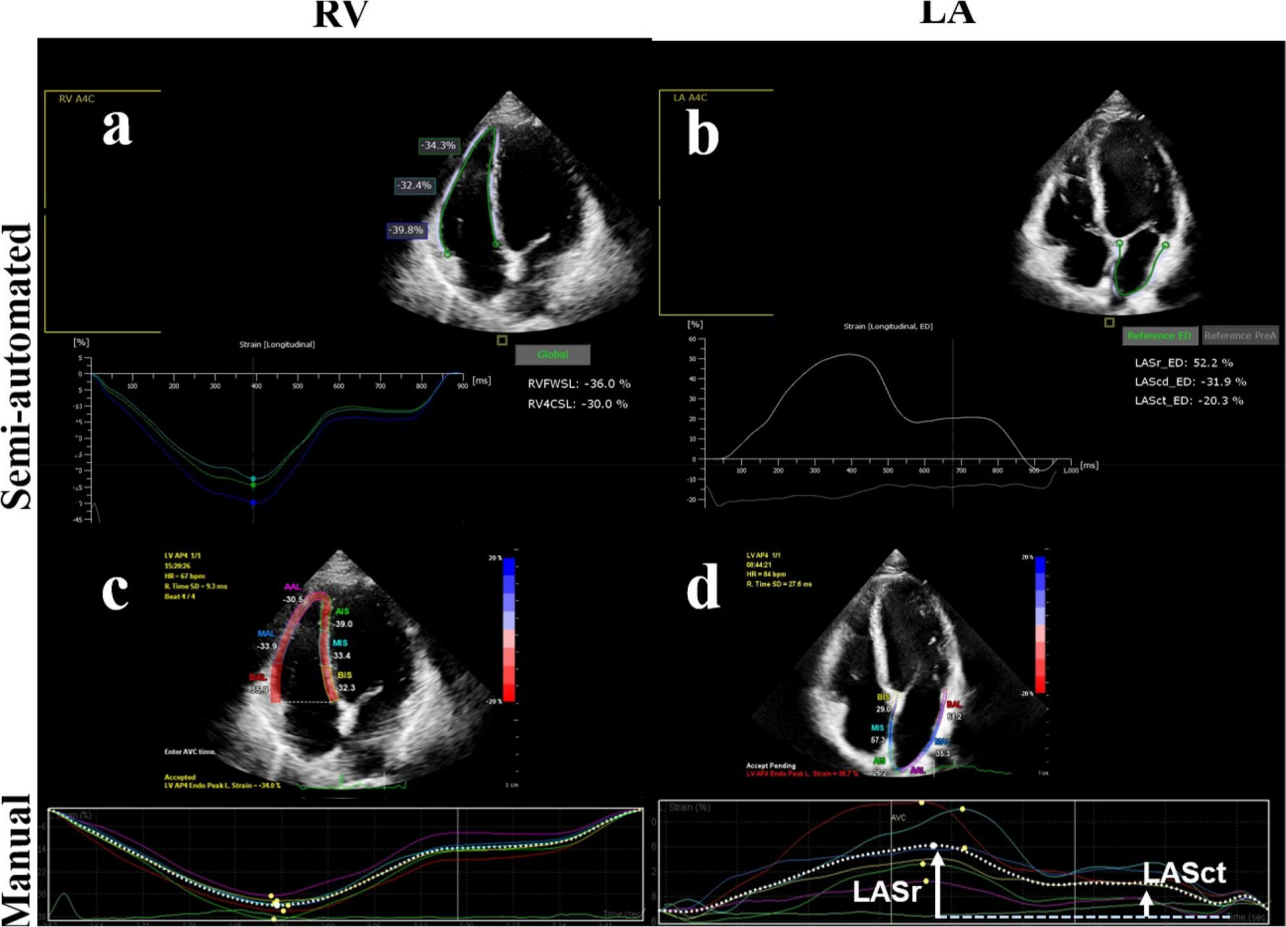
Evaluation of right ventricular free wall strain (RVFWS) and left atrial reservoir strain (LASr) by the semi-automated software (**a**, **b**) and manual (**c**, **d**) software for strain analysis. Upper panels: using the RV-specific (**a**) and LA-specific (**b**) software, the RV and LA myocardium are automatically recognized, segmented and tracked, the strain curves are displayed and the strain values calculated. Lower panels: using the manual method, the LV-specific software is used for RV (**c**) and LA (**d**) strain analysis, and the strain curve and value are obtained for each myocardial segment. The RVFWS is calculated by averaging the strain values of three RV myocardial segments

Online strain analysis was performed by the AutoStrain software. The “LV strain”, “LA strain” and “RV strain” applications were selected for the analysis of the LV, LA, and RV, respectively. The software automatically recognized the image, generated a region of interest (ROI), tracked the endocardium throughout the cardiac cycle and provided the strain values and curves for each myocardial segment (Figs. 1 and 2). After the automatic strain analysis, the two observers reviewed the tracking quality for each myocardial segment. If the tracking of more than two cardiac segments in the same view was unsatisfactory, the case was considered inadequate for analysis [6]. If necessary, each observer manually corrected the ROI to obtain a satisfactory final strain result. To assess the variability between different cardiac cycles, a different cardiac cycle was analyzed and compared with the previous one two weeks later.

### Manual strain analysis

Stored image cine-loops were assessed offline by the QLAB 9.1 Philips workstation. After the cine-loops of the 3 standard apical views were selected, observers manually defined three key points, two at the hinge points of the mitral valve annulus and one at the LV apex in each image (Fig. 1). The software then aligned and tracked the myocardium, providing segmental strain values and curves. The apical four-chamber mode used for LV strain analysis was used for both RV and LA strain analysis.

For RV strain analysis, the observer defined three points on the image, two at the lateral and septal side of the tricuspid valve annulus and one at the RV apex. The software tracked the myocardium, providing segmental and average values and curves of the RV free wall strain (RVFWS) (Fig. 2).

For the LA strain analysis, the observer defined three points on the image, two at the hinge points of the mitral valve annulus and one at the LA roof. The width of the ROI was set as 3 mm, according to guidelines [20, 21]. The LA peak strain, strain at the beginning of LA contraction, and strain at end-diastole were recorded [20]. The LA reservoir strain (LASr) was defined as LA peak strain – LA end-diastolic strain, the LA conduit strain (LAScd) was defined as the LA peak strain - strain at the beginning of atrial contraction and the LA pump strain (LASct) was defined as LA at the beginning of atrial contraction – LA end-diastolic strain (Fig. 2).

All traces were reviewed by the observers and, if necessary, they were manually corrected. At the moment of the manual strain analysis, the observer was blinded to the results of the semi-automatic strain analysis.

### Strain analysis time

In 50 randomly selected subjects the time needed to measure LV, LA, and RV strains by the two analysis methods was calculated. The strain measurement time was defined as the time between the initial selection of the echocardiographic image to analyze and the completion of the strain calculation.

### Intraobserver and interobserver variability

The intraclass correlation coefficient (ICC) and the Bland-Altman analysis were used to determine the intra-, and inter-observer reproducibility in both groups of 20 randomly selected healthy volunteers. Intraobserver variability between the first and second measurements (after 30 days) calculated by the same observer (G.P.), who was blinded to the previous measurements. Interobserver variability by two independent analysts (G.P and S.L) was calculated, with both observers were blinded to the result of the other.

### Statistical analysis

Continuous variables are expressed as the mean ± standard deviation or median and interquartile range, and categorical variables as numbers and percentages. The paired Student’s t test and linear correlation analysis were used to compare and correlate the strains measured by the two different methods. The Bland–Altman analysis was used to evaluate the agreement between the two methods and the two cardiac cycles in semi-automatic analysis. Intra- and interobserver measurement variability were assessed using the intraclass correlation coefficient (ICC) and the Bland-Altman analysis. At the Bland-Altman analysis the mean error and limits of agreement (LOAs, mean error ± 1.96 standard deviations) were calculated. The SPSS software (version 18.0) was used for statistical analysis. A p value <0.05 was considered statistically significant.

## Results

Of the 159 volunteers recruited, 3 were excluded because of severe arrythmias or moderate aortic regurgitation, and 4 due to poor image quality. A total of 152 subjects were finally enrolled (mean age 40 years, range 20–69 years). Eighty (53%) subjects were males. The mean LVEF was 62% (range 52%–73.5%). Subject characteristics are shown in Table 1.

**Table 1:** Clinical characteristics of study subjects

Variable	Study subjects (*n* = 152)
Age, years	40 ± 11
Males, n (%)	80 (53)
Height, cm	165 ± 8.2
Weight, kg	62.6 ± 11.4
Body surface area, m^2^	1.69 ± 0.18
Body mass index, kg/m^2^	22.8 ± 2.7
Heart rate, bpm	71.2 ± 10.8
Systolic blood pressure, mm Hg	113 ± 12
Diastolic blood pressure, mm Hg	75 ± 8
LVEDV, ml	110.2 ± 24.7
LVESV, ml	41.4 ± 10.8
LVEF, %	62.0 ± 7.5

*LVEDV* Left ventricular end-diastolic volume, *LVESV* Left ventricular end-systolic volume, *LVEF* Left ventricular ejection fraction

### Left ventricular strain analysis

For LVGLS measurement, the success rate of semi-automatic analysis was 95.4% (145/152) and that of manual analysis was 98.0% (149/152). In the semi-automatic analysis, 14 cases (9.6%) required manual ROI adjustment.

**Fig. 3:**
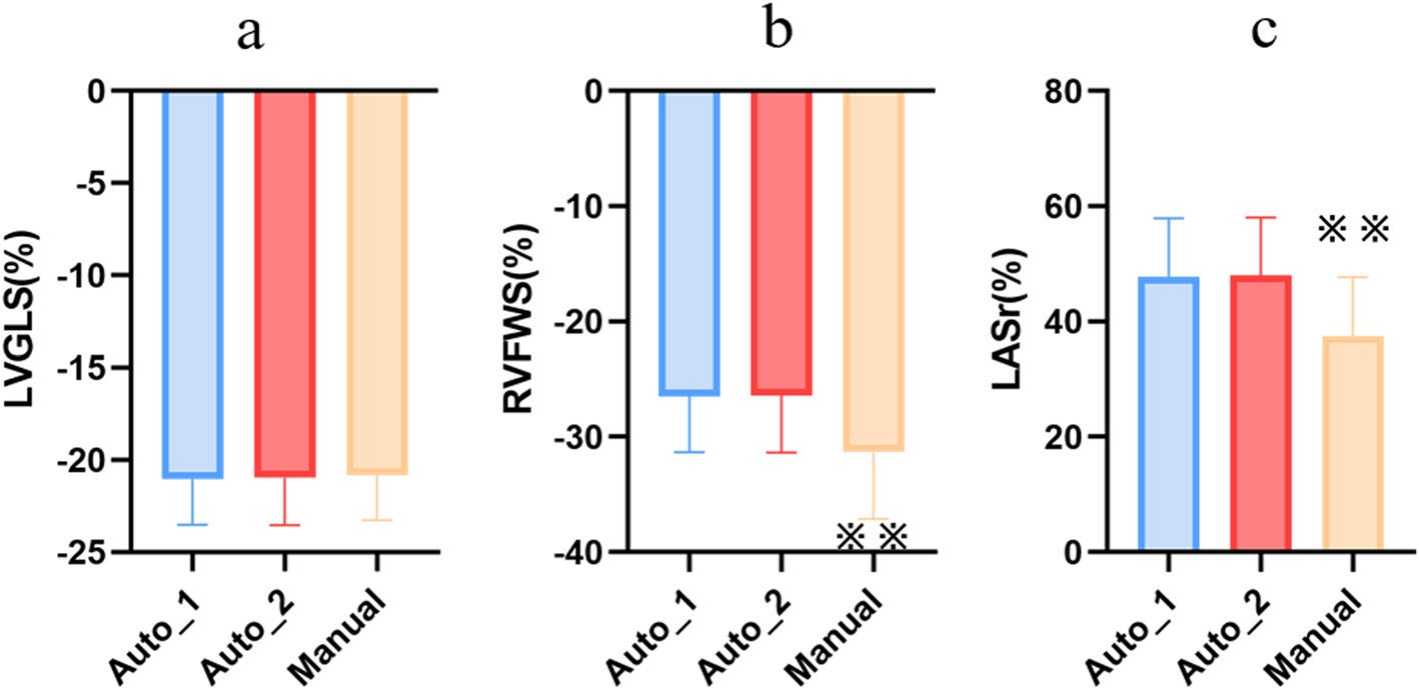
Comparison of strains assessed by semi-automatic (first cardiac cycle) vs semi-automatic (the other cardiac cycle) vs manual strain analytic software. No difference was shown in semi-automatic analysis between different cardiac cycles. Between the two methods, there were no significant differences in LVGLS (**a**). Significant differences were noted in RVFWS (**b**) and LASr (**c**). Abbreviations: auto_1, semi-automatic analysis for the first cardiac cycle; auto_2, semi-automatic analysis for the other cardiac cycle; LVGLS, left ventricular global longitudinal strain; RVFWS: right ventricular free wall strain; LASr, left atrial reservoir strain. ^※※^*p < *0.001

**Fig. 4:**
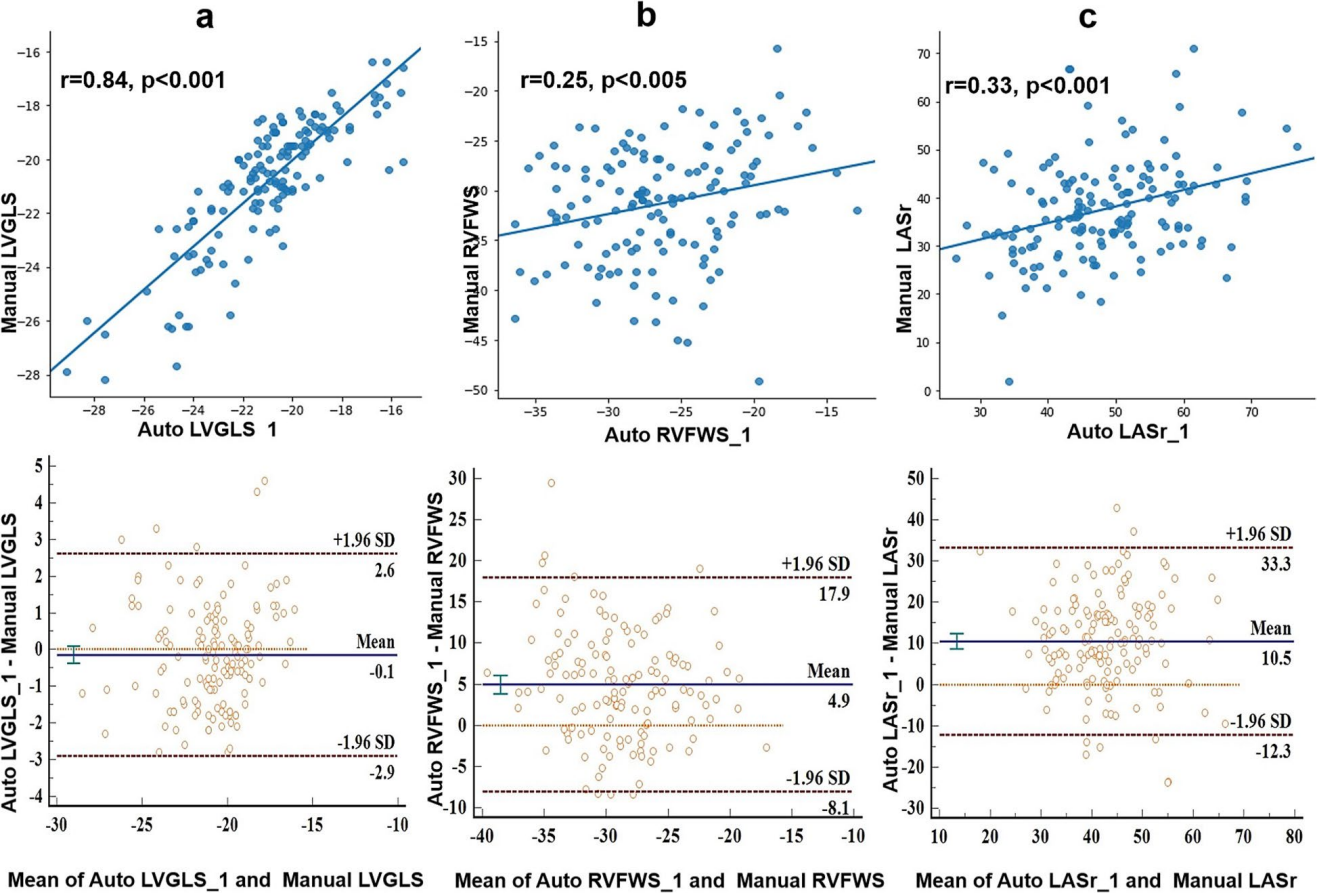
Linear regression and Bland–Altman analysis of strain measurements obtained using the semi-automatic and manual method. **a** Left ventricle global longitudinal strain (LVGLS) measurement; **b** right ventricle free wall strain (RVFWS) measurement; **c** left atrial reservoir strain (LASr) measurement. SD: standard deviation

There was no significant difference in mean value of LVGLS in semi-automatic and manual analysis (-21.0%±2.5% vs. -20.8%±2.4%, *p*=0.230; Fig. 3 and Table 2) and the correlation coefficient was 0.84 (*p*<0.001; Fig. 4). The Bland–Altman analysis showed the absence of a bias (mean error -0.1%, LOAs -2.9, 2.6%%; Fig. 4 and Table 2).

**Table 2:** Comparisons between the semi-automatic and manual strain measurements

Variable	Auto_1,%	Manual,%	p1	r	p2	Bias (LOAs)	Auto_2,%	p3	r	p4	Bias (LOAs)
LVGLS	-21.0 ± 2.5	-20.8 ± 2.4	0.230	0.84	< 0.001	-0.1(-2.9,2.6)	-21.1 ± 2.4	0.450	0.85	< 0.001	-0.1(-2.6,2.7)
RVFWS	-26.4 ± 4.8	-31.3 ± 5.8	< 0.001	0.25	< 0.005	4.9(-8.1,17.9)	-26.5 ± 4.)	0.886	0.74	< 0.001	0.1(-6.7,6.8)
LASr	48.0 ± 10.0	37.6 ± 9.9	< 0.001	0.33	< 0.001	10.5(-12.3,33.3)	47.7 ± 10.2	0.467	0.88	< 0.001	0.3(-9.4,10.0)
LAScd	-31.8 ± 8.9	-22.2 ± 7.9	< 0.001	0.55	< 0.001	-9.6(-25.4,6.2)	-31.8 ± 8.7	0.920	0.80	< 0.001	-0.0(-10.7,10.8)
LASct	-16.3 ± 6.5	-15.4 ± 6.0	0.176	0.24	< 0.005	-0.9(-16.1,14.3)	-16.0 ± 7.0	0.506	0.75	< 0.001	-0.3(-9.4,8.8)

*Abbreviations LASr* Left atrial reservoir strain, *LAScd* Left atrial conduit strain, *LASct* Left atrial pump strain, *LVGLS* Left ventricular global longitudinal strain, *RVFWS* Right ventricular free wall strain, *Manual* Manual strain measurement, *Auto_1* Semi-automatic strain measurement of the first cardiac cycle, *Auto_2* Semi-automatic strain measurement of the other cardiac cycle, *p1* Paired t-test comparing the semi-automatic and manual measurements, *p2* Linear regression assessing the relation between the semi-automatic and manual measurements, *p3* Paired t-test comparing the semi-automatic measurements of two cardiac cycles, *p4* Linear regression assessing the consistency between the semi-automatic measurements of two different cardiac cycles, *LOAs* limits of agreement

**Fig. 5:**
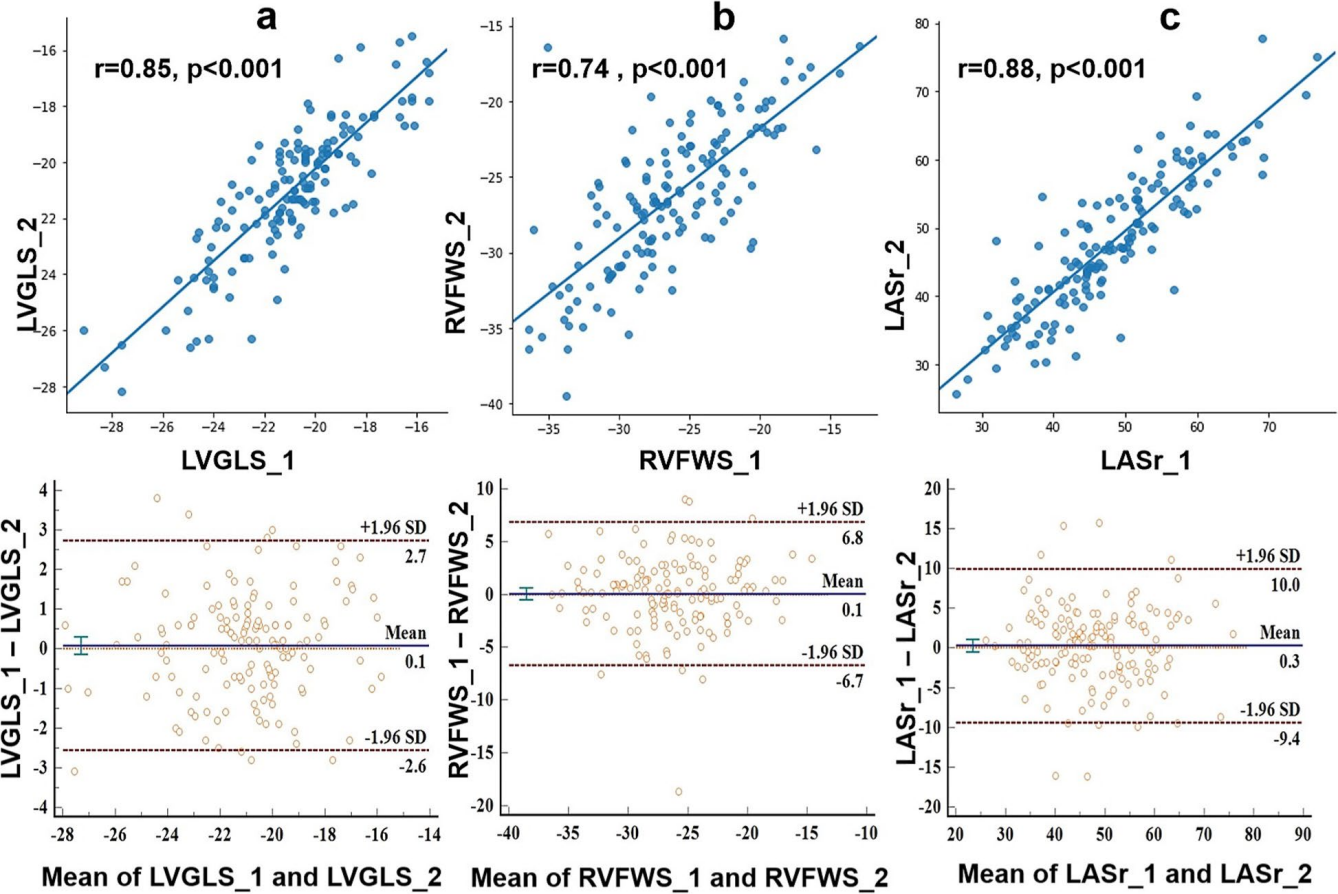
Linear correlation and Bland–Altman analysis of strain measurements between two cardiac cycles. **a** Left ventricle global longitudinal strain (LVGLS) measurement; **b** right ventricle free wall strain (RVFWS) measurement; (**c**) left atrial reservoir strain (LASr) measurement. SD: standard deviation

In semi-automatic strain analysis, there was no significant difference in LVGLS between the two cardiac cycles (-21.0%±2.5% vs. -21.1%±2.4%, *p*=0.450) (Supplementary Table 1), and correlation was good (*r*=0.85, *p*<0.001). The Bland–Altman plots are shown in Fig. 5 and reported in Table 2.

### Right ventricular free wall strain analysis

In the analysis of RVFWS, the success rate of semi-automatic strain analysis was 96.7% (147/152) and that of manual strain analysis 90.8% (138/152). In the semi-automatic strain analysis, 7 cases (4.9%) required ROI adjustment.

The semi-automatic analysis of RVFWS was significantly different from the manual strain analysis (-26.4%±4.8% vs. -31.3%±5.8%, *p*<0.001) and the absolute value of the manual strain was higher (Fig. 3 and Table 2). The correlation between the RVFWS obtained by the two methods was poor (*r*=0.248, *p*<0.005; Fig. 4). At the Bland–Altman analysis the mean error was 4.9% and the LOAs were -8.1, 17.9% (Fig. 4 and Table 2).

In semi-automatic strain analysis, there was no significant difference in RVFWS between the two cardiac cycles (-26.4%±4.8% vs. -26.5%±4.8%, *p*=0.886), and correlation was moderate (*r*=0.74, *p*<0.001; Fig. 5 and Table 2). The Bland–Altman plots are shown in Fig. 5 and reported in Table 2.

### Left atrial strain analysis

In the analysis of LA strain, the success rate of both semi-automatic and manual analysis was 99.3% (151/152). Eleven cases (7.3%) in semi-automatic strain analysis needed manual adjustment.

There were significant differences between semi-automatic and manual analysis for LASr and LAScd. In particular, the absolute value of semi-automatic strain was higher (LASr: 48.0%±10.0% vs. 37.6%±9.9%, *p*<0.001; LAScd: -31.8%±8.9% vs. -22.2%±7.9%, *p*<0.001) (Fig. 3). For LASct, there was no significant difference between the two methods (-16.3%±6.5% vs. -15.4%±6.0%, *p*=0.176).

All the LA strains obtained by the two methods were correlated. The correlation of LAScd values was moderate (*r*=0.55, *p*<0.001), but that between LASr and LASct values was poor (LASr: *r*=0.33, *p*<0.001; LASct: r=0.21, *p*<0.005) (Fig. 4). The Bland–Altman analysis showed a large bias of 10.5% with wide LOAs (-12.3, 33.3%).

In semi-automatic strain analysis, there was no significant difference in LA strain between the two cardiac cycles (LASr: 48.0%±10.0% vs. 47.7%±10.2%, *p*=0.467; LAScd: -31.8% ±8.9% vs. -31.8%±8.7%, *p*=0.920; LASct: -16.3%±6.5% vs. -16.0%±7.0%, *p*=0.506). Correlation between the two cardiac cycles were good (LASr: *r*=0.88, *p*<0.001; LAScd: *r*=0.80, *p*<0.001; LASct: *r*=0.75, *p*<0.001) (Fig. 5). The Bland–Altman plots are shown in Fig. 5 and reported in table 2.

### Comparison of strain analysis time

**Fig. 6:**
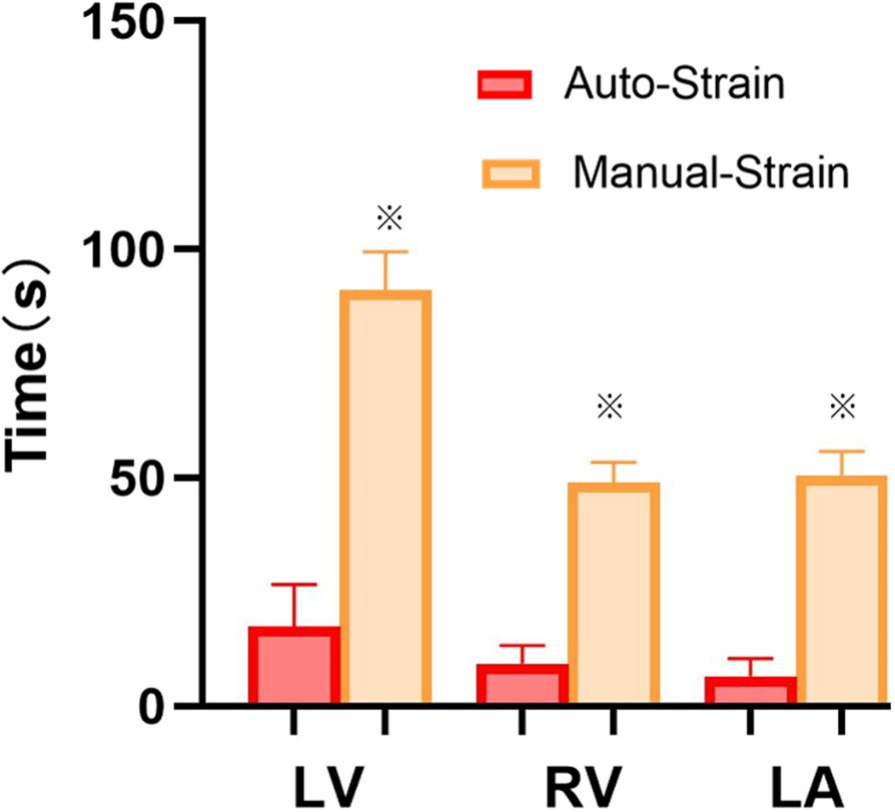
Comparison of time consumption for semi-automatic vs manual strain analysis. ^※^*p < *0.001

The average times for LV, RV, and LA strain analysis in 50 subjects were: 17 s, 9 s, and 7 s for semi-automatic analysis and 91 s, 49 s, and 51 s for manual analysis (all *p*<0.001), respectively (Fig. 6).

### Reproducibility analysis

For semi-automatic strain analysis, intra-observer and inter-observer ICCs for LVGLS, RVFWS, and LASr measurements were 0.96–0.98 and 0.90–0.95, respectively. At the Bland–Altman analysis, intra-observer mean error and LOAs were -0.08% (-1.98, 1.82%) for LVGLS, 0% (-3.5, 3.5%) for RVFWS, and 0.3% (-6.1, 6.7%) for LASr measurements; inter-observer mean error and LOAs were -0.08 (-2.68, 2.52%) for LVGLS, 0.23% (-4.67, 5.12%%) for RVFWS, and 0.5% (-7.3, 8.3%) for LASr measurements.

For manual strain analysis, intra-observer, and inter-observer ICCs for LVGLS, RVFWS, and LASr measurements were 0.82–0.90 and 0.80–0.87 (Supplementary Table 1).

## Discussion

Myocardial strain can assess myocardial function and its application is increasing in clinical practice. However, the conventional approach to myocardial strain measurement is complex, time-consuming, and dependent on the observers’ experience, thus novel methods have been developed which rely on a semi-automatic and chamber-specific analysis. In this study a novel and conventional software for myocardial strain measurements have been compared and reproducibility of the novel software has been evaluated (Graphical Abstract).

### Reproducibility analysis

Intra- and inter-observer variabilities of semi-automatic measurements of LVGLS, RVFWS and LASr were better than those of manual strain analysis of the same measurements, as indicated by higher ICC values. Also, cycle-to-cycle variability with the semi-automatic method was assessed, measuring images from two consecutive cycles with stable heart rate. At the Bland-Altman analysis the LOAs of cycle-to-cycle measurements were wider than those of intra-observer measurement, which may indicate the variability caused by different cardiac cycles.

### Left ventricular strain analysis

In our study the semi-automated LV strain analysis had a high success rate (95.4%), comparable to the feasibility of previous studies on automated strain analysis [18, 22]. Only 9.6% of cases required manual adjustments, a significantly lower percentage compared to that reported by Kawakami H et al. [22], which was close to 40%. This may be explained by the different study population (healthy volunteers vs. asymptomatic patients with heart failure risk factors).

Semi-automatic strain analysis shortens the analysis time. The most widely-used speckle tracking method currently requires several steps performed by the operator, with an execution time ranging from 5 to 10 minutes [23, 24]. Artificial intelligence based on deep learning by Zhang et al. [25] achieved semi-automatic LV strain analysis, but it still took a long time to calculate GLS of each view, which was 1 to 4 minutes. In the study of Kawakami et al. [22], the AutoStrain software was applied for automatic strain analysis of the LV, and the analysis time also required 0.5 min/patient. In this study, the time required for strain analysis of the LV was significantly shorter than that of conventional strain analysis, and it was similar to the time required for the recent automatic strain analysis of the LV based on deep learning artificial intelligence (17.4s vs 13s) [26]. This analysis time can meet the need for a rapid point-of care evaluation of critically ill patients. Compared with the manual strain analysis method (the reference method recommended by the guidelines), the results of semi-automatic strain analysis and manual strain analysis showed good reproducibility and agreement.

### Strain analysis of the right ventricular free wall

In this study we showed that RVFWS analysis had a high success rate, with a feasibility of 96.7%, which is comparable to the feasibility of previous studies [27, 28]. In some cases endocardial tracking was limited by poor image quality or by cardiac and respiratory motion, determining the RVFW to be “out of volume” in some frames (mostly in diastole).

We found that the semi-automatic analysis of the RVFWS provided different results in comparison with the manual analysis. Mirea et al. showed that the RVFWS obtained by a RV-specific tracking software was slightly lower than that based on a non-dedicated software, although the difference was not statistically significant (*p*=0.05) [29]. Li et al. showed that automated RVFWS was significantly higher than manual RVFWS in the whole study population but not in the subgroup with normal RV function [28]. These controversial results need further investigations.

The semi-automatic analysis of RVFWS required, on average, only 9 s to be completed, which is consistent with recent research [28]. Other similar RV-dedicated software needed several key points to be manually defined in the analysis process, so the average analysis time was 58 s [29]. Therefore, the automated analysis software evaluated in our study has the potential to facilitate the assessment of RV function.

### Strain analysis of the left atrium

The feasibility of the semi-automatic strain analysis of the LA in this study is relatively high, comparable to the feasibility of previous studies [18]. The LASr obtained by semi-automatic strain was significantly higher than that evaluated by manual strain: this might be the consequence of a different ROI setting. In manual LA strain analysis, the ROI width was adjusted according to guidelines, but the overall ROI was a uniform elliptic curve with a width of 3 mm, which may exceed the actual thickness of the LA wall. In addition, the wall of the LA is not completely uniform in thickness. The wall of the interatrial septum is very thin, and the motion range is large. Therefore, the movement of the interatrial septum may not be well tracked in manual method, which may cause underestimation of the overall longitudinal strain. A large ROI may lead to the inclusion of adjacent pericardium, pulmonary vein wall, and other structures, thus underestimating the strain value [30].

Mirea et al. [29] compared the LA strain obtained using a non-LA specific and a LA-specific software and found that LASr was slightly higher when using a LA-specific tool, although the difference was not statistically significant. Results of our investigation are in line with the conclusions of Mirea et al. In the study of Mirea et al., the LA dedicated tool was manual rather than semi-automated, and required to define the position of the mitral valve ring and the left atrial roof [29].

In this study, the semi-automatic analysis of LA strain improved the time-efficiency of the analysis. The analysis time of LA strain was significantly shortened compared to a conventional LA strain study [31] (7 s vs. 51 s) and to a LA specific strain analysis study [29] (7 s vs. 45 s).

## Limitations

(1) The sample size is relatively small and only healthy people with normal LVEF were included. (2) We did not provide a comparison with an external reference technique for strain measure, for example cardiac magnetic resonance imaging. This study only compared two different echocardiographic approaches to myocardial strain evaluation, thus it lacks assessment of accuracy for each method. (3) The detection of myocardial strain was based on 2D-STE and therefore has the limitations of a 2D approach.

## Conclusions

Semi-automatic strain analysis has the potential to improve efficiency in measurement of myocardial strain. It shows good agreement with the manual analysis for LV strain measurement.

## Supplementary Information

**Additional file 1:** **Supplementary Table 1.** Comparisons between the fully-automatic and manual strain measurements.

## Data Availability

The data that support the findings of this study are available on request from the corresponding author. The data are not publicly available due to privacy or ethical restrictions.

## References

[1] Trivedi SJ, Choudhary P, Lo Q, Sritharan HP, Iyer A, Batumalai V (2019). Persistent reduction in global longitudinal strain in the longer term after radiation therapy in patients with breast cancer. Radiother Oncol.

[2] Lyon AR, Lopez-Fernandez T, Couch LS, Asteggiano R, Aznar MC, Bergler-Klein J (2022). 2022 ESC Guidelines on cardio-oncology developed in collaboration with the European Hematology Association (EHA), the European Society for Therapeutic Radiology and Oncology (ESTRO) and the International Cardio-Oncology Society (IC-OS). Eur Heart J Cardiovasc Imaging.

[3] Ciarka A, Cordeiro F, Droogne W, Van Cleemput J, Voigt JU (2022). Speckle-tracking-based global longitudinal and circumferential strain detect early signs of antibody-mediated rejection in heart transplant patients. Eur Heart J Cardiovasc Imaging.

[4] De Luca A, Stolfo D, Caiffa T, Korcova R, Barbati G, Vitrella G (2019). Prognostic Value of Global Longitudinal Strain-Based Left Ventricular Contractile Reserve in Candidates for Percutaneous Correction of Functional Mitral Regurgitation: Implications for Patient Selection. J Am Soc Echocardiogr.

[5] Namazi F, van der Bijl P, Hirasawa K, Kamperidis V, van Wijngaarden SE, Mertens B (2020). Prognostic Value of Left Ventricular Global Longitudinal Strain in Patients With Secondary Mitral Regurgitation. J Am Coll Cardiol.

[6] Lang RM, Badano LP, Mor-Avi V, Afilalo J, Armstrong A, Ernande L (2015). Recommendations for cardiac chamber quantification by echocardiography in adults: an update from the American Society of Echocardiography and the European Association of Cardiovascular Imaging. Eur Heart J Cardiovasc Imaging.

[7] Donal E, Behagel A, Feneon D (2015). Value of left atrial strain: a highly promising field of investigation. Eur Heart J Cardiovasc Imaging.

[8] Mirea O, Berceanu M, Donoiu I, Militaru C, Saftoiu A, Istratoaie O (2019). Variability of right ventricular global and segmental longitudinal strain measurements. Echocardiography.

[9] Morris DA, Takeuchi M, Krisper M, Kohncke C, Bekfani T, Carstensen T (2015). Normal values and clinical relevance of left atrial myocardial function analysed by speckle-tracking echocardiography: multicentre study. Eur Heart J Cardiovasc Imaging.

[10] Frydas A, Morris DA, Belyavskiy E, Radhakrishnan AK, Kropf M, Tadic M (2020). Left atrial strain as sensitive marker of left ventricular diastolic dysfunction in heart failure. ESC heart failure.

[11] Kim J, Yum B, Palumbo MC, Sultana R, Wright N, Das M (2020). Left Atrial Strain Impairment Precedes Geometric Remodeling as a Marker of Post-Myocardial Infarction Diastolic Dysfunction. JACC Cardiovasc Imaging.

[12] Todaro MC, Carerj S, Khandheria B, Cusma-Piccione M, La Carrubba S, Antonini-Canterin F (2016). Usefulness of atrial function for risk stratification in asymptomatic severe aortic stenosis. J Cardiol.

[13] Carluccio E, Biagioli P, Lauciello R, Zuchi C, Mengoni A, Bardelli G (2019). Superior Prognostic Value of Right Ventricular Free Wall Compared to Global Longitudinal Strain in Patients With Heart Failure. J Am Soc Echocardiogr.

[14] Goedemans L, Hoogslag GE, Abou R, Schalij MJ, Marsan NA, Bax JJ (2019). ST-Segment Elevation Myocardial Infarction in Patients With Chronic Obstructive Pulmonary Disease: Prognostic Implications of Right Ventricular Systolic Dysfunction as Assessed with Two-Dimensional Speckle-Tracking Echocardiography. J Am Soc Echocardiogr.

[15] Ozawa K, Funabashi N, Tanabe N, Tatsumi K, Kobayashi Y (2016). Contribution of myocardial layers of right ventricular free wall to right ventricular function in pulmonary hypertension: Analysis using multilayer longitudinal strain by two-dimensional speckle-tracking echocardiography. Int J Cardiol.

[16] Uzan C, Lairez O, Raud-Raynier P, Garcia R, Degand B, Christiaens LP (2018). Right ventricular longitudinal strain: a tool for diagnosis and prognosis in light-chain amyloidosis. Amyloid.

[17] Negishi K, Negishi T, Kurosawa K, Hristova K, Popescu BA, Vinereanu D (2015). Practical guidance in echocardiographic assessment of global longitudinal strain. JACC Cardiovasc Imaging.

[18] Kitano T, Nabeshima Y, Negishi K, Takeuchi M. Prognostic value of automated longitudinal strain measurements in asymptomatic aortic stenosis. Heart (British Cardiac Society). 2020;107(7):318–256. 10.1136/heartjnl-2020-318256 33318081

[19] Voigt JU, Pedrizzetti G, Lysyansky P, Marwick TH, Houle H, Baumann R (2015). Definitions for a common standard for 2D speckle tracking echocardiography: consensus document of the EACVI/ASE/Industry Task Force to standardize deformation imaging. J Am Soc Echocardiogr.

[20] Badano LP, Kolias TJ, Muraru D, Abraham TP, Aurigemma G, Edvardsen T (2018). Standardization of left atrial, right ventricular, and right atrial deformation imaging using two-dimensional speckle tracking echocardiography: a consensus document of the EACVI/ASE/Industry Task Force to standardize deformation imaging. Eur Heart J Cardiovasc Imaging.

[21] Voigt JU, Mlescu GG, Haugaa K, Badano L. How to do LA strain. Eur Heart J Cardiovasc Imaging. 2020;21(7):715–7. 10.1093/ehjci/jeaa091 32548623

[22] Kawakami H, Wright L, Nolan M, Potter EL, Yang H, Marwick TH (2021). Feasibility, Reproducibility, and Clinical Implications of the Novel Fully Automated Assessment for Global Longitudinal Strain. J Am Soc Echocardiogr.

[23] Barbier P, Mirea O, Cefalu C, Maltagliati A, Savioli G, Guglielmo M (2015). Reliability and feasibility of longitudinal AFI global and segmental strain compared with 2D left ventricular volumes and ejection fraction: intra- and inter-operator, test-retest, and inter-cycle reproducibility. Eur Heart J Cardiovasc Imaging.

[24] Manovel A, Dawson D, Smith B, Nihoyannopoulos P (2010). Assessment of left ventricular function by different speckle-tracking software. Eur J Echocardiogr.

[25] Zhang J, Gajjala S, Agrawal P, Tison GH, Hallock LA, Beussink-Nelson L (2018). Fully Automated Echocardiogram Interpretation in Clinical Practice. Circulation.

[26] Salte IM, Ostvik A, Smistad E, Melichova D, Nguyen TM, Karlsen S (2021). Artificial Intelligence for Automatic Measurement of Left Ventricular Strain in Echocardiography. JACC Cardiovasc Imaging.

[27] Muraru D, Onciul S, Peluso D, Soriani N, Cucchini U, Aruta P (2016). Sex- and Method-Specific Reference Values for Right Ventricular Strain by 2-Dimensional Speckle-Tracking Echocardiography. Circ Cardiovasc Imaging.

[28] Li Y, Sun C, Zhang L, Zhang Y, Wang J, Zhang J (2022). Feasibility, Reproducibility, and Prognostic Value of Fully Automated Measurement of Right Ventricular Longitudinal Strain. J Am Soc Echocardiogr.

[29] Mirea O, Duchenne J, Voigt JU (2022). Comparison between Nondedicated and Novel Dedicated Tracking Tool for Right Ventricular and Left Atrial Strain. J Am Soc Echocardiogr.

[30] Spriestersbach H, Oh-Ici D, Schmitt B, Berger F, Schmitz L (2015). The influence of the region of interest width on two-dimensional speckle tracking-based measurements of strain and strain rate. Echocardiography.

[31] Cameli M, Caputo M, Mondillo S, Ballo P, Palmerini E, Lisi M (2009). Feasibility and reference values of left atrial longitudinal strain imaging by two-dimensional speckle tracking. Cardiovasc Ultrasound.

