# Supplementary material for: Feasibility and reproducibility of semi-automated longitudinal strain analysis: a comparative study with conventional manual strain analysis
Source: Cardiovasc Ultrasound. 2023 Jul 19;21:12. doi: 10.1186/s12947-023-00309-5 (PMC10355018; doi:10.1186/s12947-023-00309-5)
Supplement: Supplementary file 1 — Additional file 1: Supplementary Table 1. Comparisons between the fully-automatic and manual strain measurements. [file 12947_2023_309_MOESM1_ESM.docx]

**Supplementary Materials**


**Supplementary Table 1 Comparisons between the fully-automatic and manual strain measurements**

| **Variable** | **Intra-observer ICC** | | **Inter-observer ICC** | |
| --- | --- | --- | --- | --- |
|  | Manual | Automatic | Manual | Automatic |
| LVGLS | 0.90 ^※^  (0.78-0.96) | 0.98 ^※^  (0.94-0.99) | 0.87 ^※^  (0.70-0.95) | 0.95^※^  (0.86-0.98) |
| RVFWS | 0.87 ^※^  (0.70-0.95) | 0.96 ^※^  (0.90-0.99) | 0.84 ^※^  (0.64-0.93) | 0.92 ^※^  (0.80-0.97) |
| LASr | 0.82^※^  (0.60-0.92) | 0.97 ^※^  (0.93-0.99) | 0.80 ^※^  (0.57-0.92) | 0.90 ^※^  (0.77-0.96) |

*Note:* ICC, intra-class correlation coefficient

^※^p＜0.001.
